# Simulation of pooled-sample analysis strategies for COVID-19 mass testing

**DOI:** 10.2471/BLT.20.257188

**Published:** 2020-07-06

**Authors:** Andreas Deckert, Till Bärnighausen, Nicholas NA Kyei

**Affiliations:** a Heidelberg Institute of Global Health, Heidelberg University, Im Neuenheimer Feld 324, 69120, Heidelberg, Germany.

## Abstract

**Objective:**

To evaluate two pooled-sample analysis strategies (a routine high-throughput approach and a novel context-sensitive approach) for mass testing during the coronavirus disease 2019 (COVID-19) pandemic, with an emphasis on the number of tests required to screen a population.

**Methods:**

We used Monte Carlo simulations to compare the two testing strategies for different infection prevalences and pooled group sizes. With the routine high-throughput approach, heterogeneous sample pools are formed randomly for polymerase chain reaction (PCR) analysis. With the novel context-sensitive approach, PCR analysis is performed on pooled samples from homogeneous groups of similar people that have been purposively formed in the field. In both approaches, all samples contributing to pools that tested positive are subsequently analysed individually.

**Findings:**

Both pooled-sample strategies would save substantial resources compared to individual analysis during surge testing and enhanced epidemic surveillance. The context-sensitive approach offers the greatest savings: for instance, 58–89% fewer tests would be required for a pooled group size of 3 to 25 samples in a population of 150 000 with an infection prevalence of 1% or 5%. Correspondingly, the routine high-throughput strategy would require 24–80% fewer tests than individual testing.

**Conclusion:**

Pooled-sample PCR screening could save resources during COVID-19 mass testing. In particular, the novel context-sensitive approach, which uses pooled samples from homogeneous population groups, could substantially reduce the number of tests required to screen a population. Pooled-sample approaches could help countries sustain population screening over extended periods of time and thereby help contain foreseeable second-wave outbreaks.

## Résumé

**Objectif:**

Évaluer deux stratégies d'analyse par échantillons groupés (une approche de routine à haut débit et une nouvelle approche contextuelle) en vue d'instaurer un dépistage de masse durant la pandémie de maladie à coronavirus 2019 (COVID-19), en mettant l'accent sur le nombre de tests requis pour dépister la population.

**Méthodes:**

Nous avons utilisé des simulations Monte-Carlo afin de comparer les deux stratégies de dépistage pour différentes prévalences d'infection et groupes d'échantillons de différentes tailles. Avec l'approche de routine à haut débit, des groupes d'échantillons hétérogènes sont formés aléatoirement pour subir un test de réaction en chaîne par polymérase (PCR). Avec la nouvelle approche contextuelle, le test PCR est effectué sur des échantillons groupés provenant d'ensembles homogènes de personnes similaires, qui ont été formés à cette intention sur le terrain. Dans ces deux approches, tous les échantillons du groupe qui se sont avérés positifs sont ensuite analysés séparément.

**Résultats:**

Les deux stratégies par échantillons groupés permettraient de ménager d'importantes ressources par rapport aux analyses individuelles lorsqu'un dépistage de masse est nécessaire et qu'il faut accroître la surveillance de l'épidémie. L'approche contextuelle est celle qui permet de faire le plus d'économies: le nombre de tests requis diminuerait par exemple de 58 à 89% pour un groupe de 3 à 25 échantillons recueillis au sein d'une population de 150 000 personnes avec une prévalence d'infection de 1% ou 5%. Proportionnellement, l'approche de routine à haut débit demanderait 24 à 80% de tests en moins qu'un dépistage individuel.

**Conclusion:**

Les tests PCR sur des échantillons groupés permettraient d'optimiser les ressources lors d'un dépistage de masse dans le cadre de la pandémie de COVID-19. En particulier la nouvelle approche contextuelle, qui se base sur des échantillons groupés provenant de groupes de personnes homogènes et pourrait réduire considérablement le nombre de tests nécessaire pour dépister une population. Grâce aux analyses par échantillons groupés, les pays pourraient continuer à tester la population sur de longues périodes et contribuer ainsi à contenir les prochaines flambées épidémiques.

## Resumen

**Objetivo:**

Evaluar dos estrategias en el análisis de muestras colectivas (un método de rutina de alto rendimiento y un método innovador sensible al contexto) para realizar pruebas en masa durante la pandemia de la enfermedad del coronavirus 2019 (COVID-19), prestando especial atención al número de pruebas que se requieren para examinar a una población.

**Métodos:**

Se aplicaron simulaciones de Monte Carlo para comparar las dos estrategias de prueba para distintas prevalencias de infección y tamaños combinados de grupos. Mediante el método de rutina de alto rendimiento, se forman grupos de muestras heterogéneas de manera aleatoria para analizar la reacción en cadena de la polimerasa (PCR, por sus siglas en inglés). Mediante el método innovador sensible al contexto, el análisis PCR se realiza con muestras colectivas de grupos homogéneos de personas similares que se han formado intencionadamente en la zona. Al aplicar ambos métodos, todas las muestras que participan en los grupos con resultados positivos se analizan posteriormente de forma individual.

**Resultados:**

Ambas estrategias de muestreo colectivo ahorrarían recursos significativos en comparación con los análisis individuales durante un aumento repentino de las pruebas, y mejorarían la vigilancia de la epidemia. El método sensible al contexto ofrece el mayor ahorro: por ejemplo, se necesitarían entre un 58 % y un 89 % menos de pruebas para un grupo colectivo de 3 a 25 muestras en una población de 150 000 habitantes con una prevalencia de la infección del 1 % o el 5 %. Asimismo, la estrategia de alto rendimiento de rutina requeriría entre un 24 % y un 80 % menos de pruebas que las pruebas individuales.

**Conclusión:**

La prueba PCR de muestra colectiva podría ahorrar recursos durante las pruebas en masa de la COVID-19. En especial, el método innovador sensible al contexto, que emplea muestras colectivas de grupos de población homogéneos, podría reducir significativamente el número de las pruebas que se requieren para examinar a una población. Los métodos de muestras colectivas podrían ayudar a los países a mantener el cribado de la población durante periodos de tiempo prolongados y, de ese modo, ayudar a contener los previsibles brotes de una segunda oleada.

## ملخص

الغرض تقييم استراتيجيتين لتحليل العينات المجمعة (نهج روتيني عالي الإنتاجية ونهج مستجد حساس للسياق) للاختبار الشامل خلال المرض الوبائي كورونا لعام 2019 (كوفيد-19)، مع التركيز على عدد الاختبارات المطلوبة لفحص السكان.

الطريقة استخدمنا أساليب محاكاة مونت كارلو لمقارنة استراتيجيتي الاختبار لنسب انتشار العدوى المختلفة، وأحجام المجموعة المجمعة. مع النهج الروتيني عالي الإنتاجية، يتم تشكيل مجموعات العينات غير المتجانسة بشكل عشوائي لتحليل تفاعل سلسلة بوليميريز (PCR). باستخدام النهج المستجد القائم على السياق، يتم إجراء تحليل PCR على عينات مجمعة من مجموعات متجانسة من أشخاص متشابهين تم تكوينهم عن قصد في هذا المجال. في كلا النهجين، فإن كل العينات التي ساهمت في المجمعات التي ثبت بعد الاختبار أنها إيجابية، تم لاحقاً تحليلها بشكل فردي.

النتائج توفر كل من إستراتيجيتي العينات المجمعة موارد هائلة عند مقارنتها بالتحليل الفردي أثناء اختبار الطفرة ومراقبة الأوبئة المحسنة. يوفر النهج القائم على السياق أكبر قدر من التوفير: على سبيل المثال، عدد أقل من الاختبارات بنسبة 58 إلى 89٪ سيكون مطلوباً لمجموعة مجمعة بحجم من 3 إلى 25 عينة في عدد سكان يبلغ 150000 نسمة، مع انتشار للعدوى بنسبة 1% أو 5%. وعلى نحو مقابل، فإن الاستراتيجية الروتينية عالية الإنتاج تتطلب اختبارات أقل بنسبة 24 إلى 80% من الاختبارات الفردية.

الاستنتاج إن فحص PCR للعينات المجمعة قد يؤدي لتوفير الموارد أثناء الاختبار الجماعي لكوفيد-19. وبشكل خاص، فإن النهج المستجد القائم على السياق، والذي يستخدم عينات مجمعة من مجموعات سكانية متجانسة، قد يقلل بشكل كبير من عدد الاختبارات المطلوبة لفحص مجتمع سكاني. يمكن أن تساعد مناهج العينة المجمعة الدول في المداومة على فحص السكان عبر فترات طويلة من الزمن، وبالتالي المساعدة في احتواء حالات تفشي الموجة الثانية المتوقعة.

## 摘要

**目的:**

旨在评估在 2019 冠状病毒病 (COVID-19) 大流行期间用于大规模检测的两种综合样本分析策略（常规高通量法和新型背景敏感法），强调进行人群筛查时所需实施的检测量。

**方法:**

我们通过使用蒙特卡洛模拟法，比较适用于不同感染率和群体综合规模的两种检测策略。若使用常规高通量法，可随机形成异质样本池，以便执行聚合酶链反应 (PCR) 分析。若使用新型背景敏感法，则可在现场有目的地将相似的人员组建为同质群组，然后基于从同质群组处获得的综合样本执行 PCR 分析。在采用这两种方法的情况下，随后需单独分析检测结果为阳性的样本池包含的所有样本。

**结果:**

与检测数量激增和疫情监测加强期间执行的个量分析相比，两种综合样本策略均可节省大量资源。背景敏感法可最大程度地节省费用：例如，针对感染率为 1％ 或 5％ 的 150,000 人进行检测，每组样本合计数为 3 至 25 个样本，则需要执行的检测量将减少 58-89％。相应地，与逐个检测相比，常规高通量策略可将检测量减少 24–80％。

**结论:**

在大规模检测 2019 冠状病毒病期间，综合样本 PCR 筛查可节省资源。尤其是，通过使用取自同质人群的综合样本，新型背景敏感法可大幅减少筛查人群所需的检测量。综合样本法可帮助各国在较长时期内持续进行人口筛查，从而有助于遏制可预见的第二波疫情爆发。

## Резюме

**Цель:**

Оценить две стратегии анализа с применением объединенных образцов (стандартный подход с высокой пропускной способностью и новый подход с учетом контекста) применительно к массовому тестированию во время пандемии, вызванной коронавирусной инфекцией 2019 (COVID-19), с уделением особого внимания количеству тестов, необходимому для скрининга популяции.

**Методы:**

Авторы использовали моделирование методом Монте-Карло для сравнения двух различных стратегий при разных уровнях распространенности инфекции и разных размерах объединенных групп. При стандартном подходе с высокой пропускной способностью пулы разнородных образцов для проведения анализа методом полимеразной цепной реакции (ПЦР) формировались случайным образом. При новом подходе с учетом контекста анализ ПЦР выполнялся на объединенных образцах, полученных от однородных групп людей со сходными качествами, которые специально формировались на местах. В обоих подходах все входящие в пул образцы, давшие положительный результат, впоследствии подвергались индивидуальному анализу.

**Результаты:**

Обе стратегии работы с объединенными образцами позволяют значительно экономить ресурсы в сравнении с проведением индивидуальных анализов при тестировании в условиях резкого роста заболеваемости и усиленного эпидемиологического надзора. Подход с учетом контекста позволял достичь наибольшей экономии. Например, в объединенной группе размерами от 3 до 25 образцов в популяции размером 150 000 человек при уровне распространенности инфекции от 1 до 5% требовалось на 58–89% меньше тестов. Соответственно, стандартная стратегия с высокой пропускной способностью требовала на 24–80% меньше тестов в сравнении с индивидуальным анализом.

**Вывод:**

Скрининговое обследование методом ПЦР на основе объединенных образцов может способствовать экономии ресурсов в ходе массового тестирования на COVID-19. В частности, новый подход с учетом контекста, в котором используются объединенные образцы, полученные в однородных популяционных группах, может значительно снизить количество тестов, необходимых для скрининга популяции. Подходы на основе использования объединенных образцов могут помочь странам поддерживать скрининговое обследование популяции в течение длительного времени и тем самым сдержать прогнозируемые вспышки второй волны.

## Introduction

The incubation period of coronavirus disease 2019 (COVID-19) can be as long as 14 days and an unknown proportion of asymptomatic carriers is capable of transmitting the infection, these two factors present substantial challenges for controlling and mitigating the disease.^1^^–^^6^ Although around 80% of people with COVID-19 are reported to have mild disease,^7^ the remaining 20% often have severe symptoms and could potentially overwhelm health-care facilities that are already overstretched.^8^ Consequently, most countries aim to avoid large surges in patients with COVID-19 and to level the demand for health care, particularly for intensive care beds for patients with respiratory failure.^9^ Severely affected nations in the northern hemisphere have adopted drastic containment measures, including the complete lockdown of regions and countries.

As of March 2020, few cases have been reported in Africa or Latin America. However, researchers predicted that Africa would face importation and spread of COVID-19.^10^^,^^11^ Although most countries in sub-Saharan Africa are screening targeted travellers, this has proven ineffective due to the disease’s natural history, specifically the potential for spread during the incubation period. Unless swift and collective interventions are instituted, the effect of COVID-19 might be devastating for countries with fragile health systems.^11^ As a consequence, the World Health Organization (WHO) recommended that countries, particularly those experiencing their few first cases of COVID-19, should perform active surveillance, including testing, isolating cases and tracing contacts.^9^

It is highly unlikely that infection transmission will be eliminated in the next few months in countries with well-established outbreaks. Instead, the epidemic will predominantly be controlled, which will lead to the stepwise withdrawal of restrictions, albeit with localized flare-ups that could necessitate the return of strict containment measures. In second-wave outbreaks, comprehensive, rapid and cost–effective, localized mass testing may be required to identify both symptomatic and asymptomatic cases and prevent further spread. 

In both scenarios, settings with a few first cases and second-wave outbreaks, all symptomatic and asymptomatic cases of COVID-19 must be identified rapidly. Confirmation of infection, particularly in asymptomatic individuals, relies on real-time polymerase chain reaction (RT–PCR) tests for severe acute respiratory syndrome coronavirus 2 (SARS-CoV-2).^4^ Although RT–PCR tests have been used in epidemiological studies,^12^^,^^13^ they are time-consuming and costly. However, mass testing is important for a wide-range of COVID-19 control strategies and evidence of its effectiveness in a local population has been reported in the small Italian town of Vo', which has around 3000 inhabitants.^14^^,^^15^ After isolation of the approximately 3% of the population who tested positive, transmission ceased and only six individuals were still infected after 14 days. In larger populations, however, such comprehensive surveillance may be impracticable or too costly and the test workload may rapidly outstrip capacity and resources.^16^ Many low- or middle-income countries with constrained resources will find it even more difficult to carry out extensive testing and long-term lockdowns may not be an option because economic necessity could preclude self-isolation.

An established way of conserving resources during surge testing and disease outbreaks is pooled-sample analysis.^16^^–^^19^ With current pooling strategies (e.g. high-throughput, pooled, PCR testing and highly-automated, matrix, sample pooling),^16^^,^^18^^–^^22^ extracts from a random number of samples from a heterogeneous population group are combined into a single tube for pooled PCR analysis. These strategies have been shown to be cost–effective during mass testing compared with individual testing.^18^^,^^19^^,^^23^ Recent research on establishing the optimal pool size that maintains the testing accuracy for SARS-CoV-2 PCR assays has found that accuracy is retained in a pool size of up to 32 samples.^22^^,^^24^^,^^25^ It appears that costs can be reduced substantially without sacrificing accuracy.

The aims of this study were to evaluate the performance and resource needs of two pooled-sample analysis strategies for the mass-testing of SARS-CoV-2 infection during the current COVID-19 pandemic and to investigate how infection prevalence influences the optimum number of samples that can be pooled and, therefore, the number of tests required. The two strategies evaluated were: (i) routine, high-throughput, two-step, pooled-sample PCR analysis involving heterogeneous sample pools (hereafter referred to as the routine high-throughput approach); and (ii) a novel approach involving pools derived from homogeneous population groups that are purposively formed in the field (hereafter referred to as the context-sensitive approach).^22^^,^^24^^,^^25^

With the routine high-throughput approach, first sample pools are composed randomly in the laboratory for analysis. Then, in a second step, all samples that contributed to any pool that tested positive for SARS-CoV-2 are analysed individually.^16^^,^^18^^–^^21^^,^^23^^,^^26^ However, during COVID-19 outbreaks, there is a high likelihood that some members of homogeneous groups (e.g. families, office colleagues or neighbours) will become infected once one individual has imported the infection into the group. Response teams carrying out contact tracing could take advantage of this situation and designate homogenous groups in the field for subsequent pooled-sample analysis. With the context-sensitive approach, first groups of similar people of a defined size are formed and swab tests of all group members undergo pooled-sample RT–PCR analysis. Again, in the second step, all members of any group whose pooled sample tested positive are investigated individually. This second approach could require an even lower number of tests than routine high-throughput testing, thereby reducing both costs and the workforce needed for population screening.

## Methods

The cost–effectiveness of pooled-sample PCR screening is commonly assessed using computer simulations.^27^^–^^29^ For our comparison of the number of tests required with two mass testing strategies, we applied Monte Carlo simulation techniques because of the wide range of uncertainty in some parameters during the current COVID-19 pandemic.^30^^,^^31^

### Routine high-throughput approach

We investigated the performance of the routine high-throughput approach to pooled-sample analysis in two populations of 150 000 and 15 000, respectively, for a SARS-CoV-2 infection prevalence ranging from 0.5–20%, in incremental steps of 0.5%. We varied the group size from 2 to 100; correspondingly, the number of groups in a population of 150 000 varied from 75 000 to 1500, respectively. To simulate the spread of the infection, we first formed the groups and then determined the number of infected individuals within each group by applying a binomial distribution (parameters: overall prevalence and group size). The total number of tests required was the sum of the number of pooled groups (in the first step, all groups were tested) and the number of groups that tested positive times the group size (in the second step, all members of groups that tested positive were tested individually). The results of the simulation are presented as a three-dimensional graph that shows how the number of tests required varies with group size and infection prevalence. As we used stochastic variables, the surface of the plot contained some small-scale ripples, which we smoothed using a spline smoothing function. All simulations were conducted in SAS 9.4 TS1M4 (SAS Institute Inc., Cary, United States of America).

### Novel context-sensitive approach

**Fig. 1:**
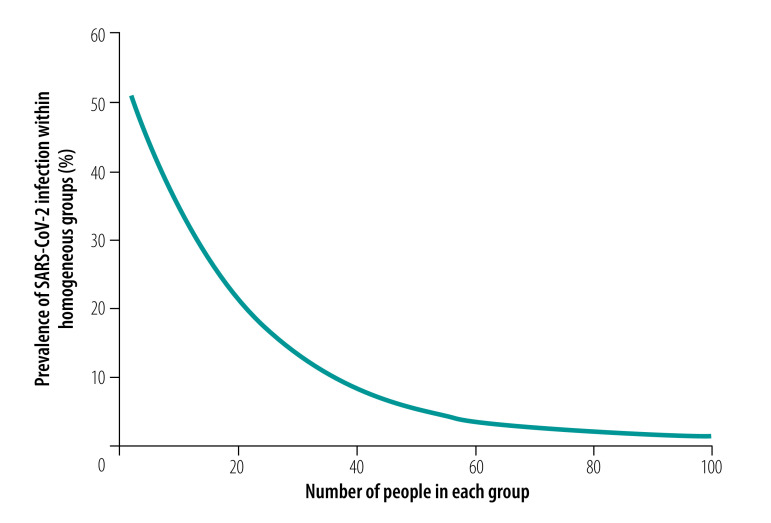
Assumed relationship between SARS-CoV-2 infection prevalence in pooled homogeneous groups and group size for a population prevalence of 0.5%, context-sensitive approach to pooled-sample analysis during a COVID-19 outbreak

COVID-19: coronavirus disease 2019; SARS-CoV-2: severe acute respiratory syndrome coronavirus 2.

Notes: The context-sensitive approach involved analysing pooled samples from groups of similar people of a defined size for real-time polymerase chain reaction testing for SARS-CoV-2. Although the graph shows a continuous variation, in the simulation both prevalence and group size were varied in discrete steps.

We repeated the simulation for the context-sensitive approach with homogeneous pooled samples. With homogeneous groups, it is reasonable to assume that the within-group variation in any characteristic is smaller than the between-group variation. Hence, if one member of a pooled group is infected with SARS-CoV-2, there is a high likelihood that other group members are also infected. In addition, we assumed that the within-group infection prevalence decreases nonlinearly with increasing group size because the composition of the group becomes more diverse as it gets larger. This relationship was assumed to be:
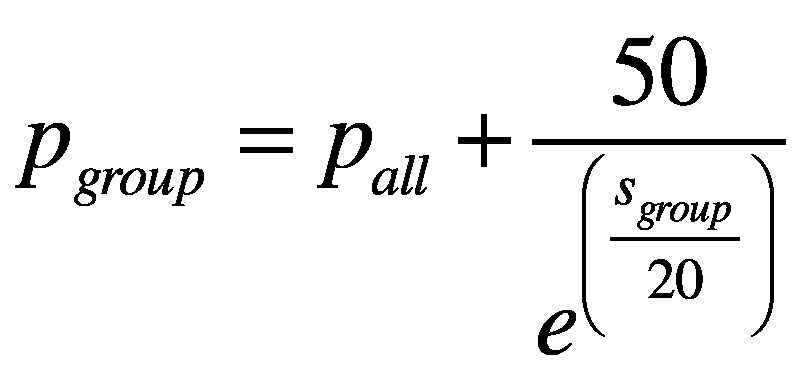
(1)where *p_group_* is the average within-group prevalence expressed as a percentage, *p_all_* is the percentage overall prevalence and *s_group_* is the size of the pooled group. For example, Fig. 1 shows the relationship between the within-group prevalence and group size for an overall prevalence of 0.5%. For a given overall prevalence, we calculated how many pooled groups would test positive for different group sizes and within-group prevalences. Furthermore, we performed a Bernoulli experiment for each group (parameter: probability that a group will test positive). Subsequently, we estimated the number of people who would test positive in each pooled group that tested positive using binomial distributions (parameters: within-group prevalence and group size). As a control measure, we calculated the overall prevalence from simulation data and found that there was a negligible difference from the initial assumed overall prevalence (available in the data repository).^32^ The other steps in the simulation were the same as those for the routine high-throughput approach. 

For the context-sensitive approach, we also performed a sensitivity analysis by determining how the number of tests saved would be affected by altering the functional form of the relationship between the within-group prevalence and the size of the homogeneous groups. In addition, to account for actual variations in group size (e.g. for households, offices in a company or seat rows in an aircraft), we investigated a scenario in which 20% of groups had two members, 30% had three members, 25% had four members, 15% had five members and 10% had six members.

For the two approaches, we calculated the percentage reduction in, and a reduction factor for, the number of tests required relative to individual sample analysis for different group sizes and for an infection prevalence of 1 and 5%. Here, we did not apply a smoothing function. The reduction factor provides another way of looking at resource savings that might be understood more intuitively than a percentage. For a population size *N_p_*, the reduction factor, *RF*, was defined as:
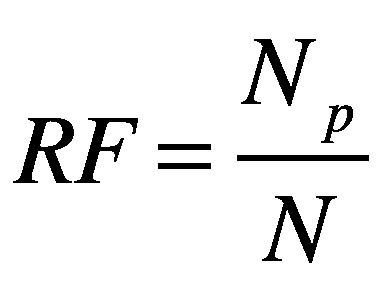
(2)where *N* is the number of tests required with the pooled-sample approach.

## Results

### Routine high-throughput approach

**Fig. 2:**
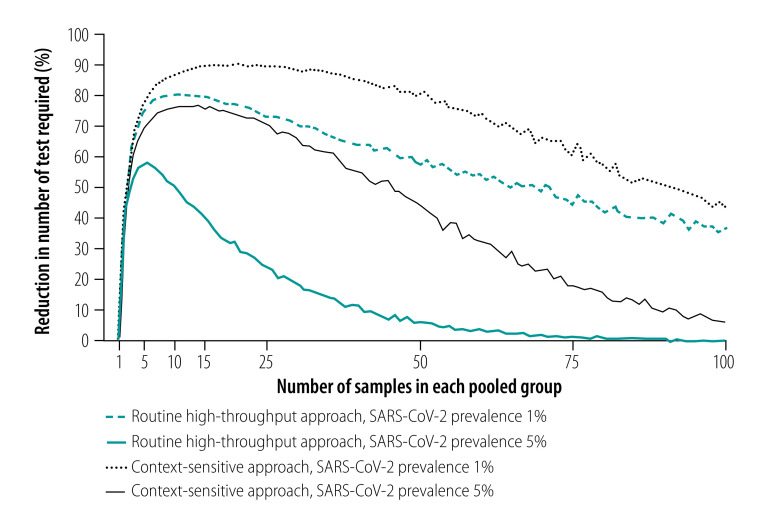
Reduction in number of tests required with pooled-sample analysis relative to individual testing during a COVID-19 outbreak, by analysis strategy, pooled group size and SARS-CoV-2 infection prevalence

COVID-19: coronavirus disease 2019; SARS-CoV-2: severe acute respiratory syndrome coronavirus 2.

Notes: The routine high-throughput approach involved analysing pooled samples from heterogeneous groups of people of a defined size for real-time polymerase chain reaction testing for SARS-CoV-2. The context-sensitive approach involved analysing pooled samples from groups of similar people of a defined size. The reduction in the number of tests required is relative to the number needed to test all individuals. Our simulation considered a population of 150 000. Although the graph shows a continuous variation in tests required, in the simulation group size was varied in discrete steps.

**Fig. 3:**
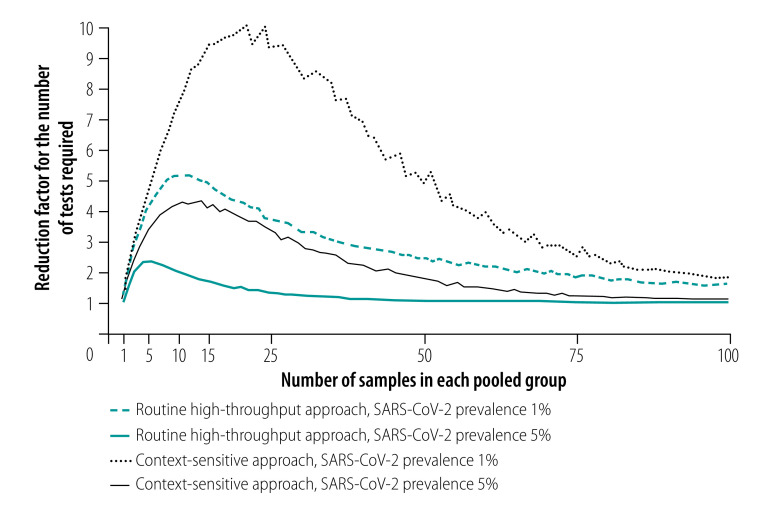
Reduction factor for the number of tests required with pooled-sample analysis relative to individual testing during a COVID-19 outbreak, by analysis strategy, pooled group size and SARS-CoV-2 infection prevalence

COVID-19: coronavirus disease 2019; SARS-CoV-2: severe acute respiratory syndrome coronavirus 2.

Notes: The routine high-throughput approach involved analysing pooled samples from heterogeneous groups of people of a defined size for real-time polymerase chain reaction testing for SARS-CoV-2. The context-sensitive approach involved analysing pooled samples from groups of similar people of a defined size. The reduction factor was defined as the number of tests required for individual testing of a population divided by the number of tests required in the same population using a pooled-sample approach. Our simulation considered a population of 150 000. Although the graph shows a continuous variation in tests required, in the simulation group size was varied in discrete steps.

**Fig. 4:**
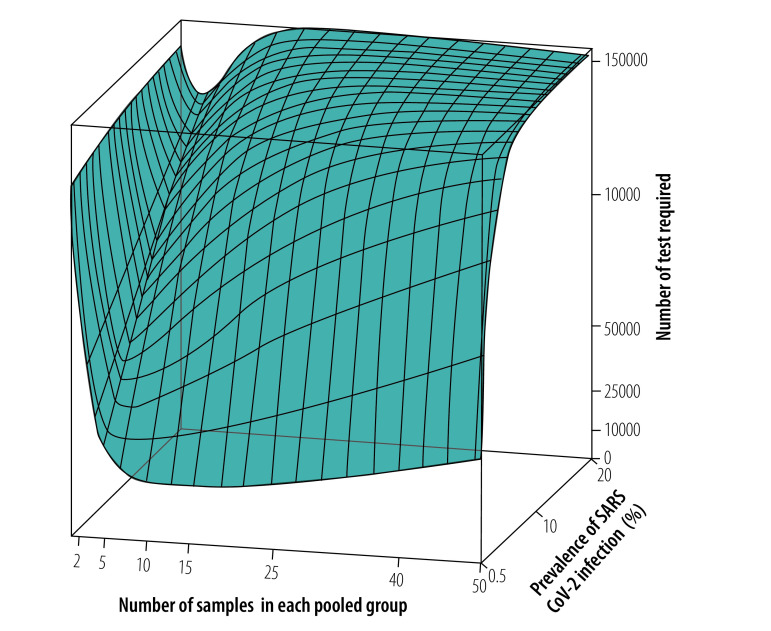
Surface plot of tests required using a routine high-throughput approach to pooled-sample analysis, by pooled group size and SARS-CoV-2 infection prevalence

As expected, the analysis showed that the number of tests required increased as the prevalence of infection increased.^29^ For an overall infection prevalence of 1 or 5%, the number of tests required with the routine high-throughput approach was 24–80% less than with individual sample analysis for group sizes of 3 to 25 in a population of 150 000 (Fig. 2 and data repository).^32^ The corresponding reduction factors are shown in Fig. 3. Given this low prevalence, a substantial reduction in tests required can be achieved with a wide range of group sizes. For example, with a prevalence of 1%, selecting a group size between 5 and 50 implies at least 58% fewer tests. With a high prevalence of 10%, a reduction in the number of tests of around 40% can still be achieved but the selected group size must be close to 3 (data repository).^32^ When the prevalence is high and the group size is large, the number of tests required slightly exceeds the number required for individual testing. Fig. 4 shows the number of tests required with the routine high-throughput approach for a wide range of prevalences and groups sizes in a population of 150 000. The minimum number of tests required in this population was 20 388, which was achieved when the prevalence was 0.5% and the group size was 14. This result corresponded to 86% (129 612/150 000) fewer tests and a reduction factor of 7.4 compared with individual testing. The surface plot for a population of 15 000 was similar.^32^

SARS-CoV-2: severe acute respiratory syndrome coronavirus 2.

Notes: The routine high-throughput approach involved analysing pooled samples from heterogeneous groups of people of a defined size for real-time polymerase chain reaction testing for SARS-CoV-2. Our simulation considered a population of 150 000. Although the figure shows a continuous variation in tests required, in the simulation both prevalence and group size were varied in discrete steps.

### Novel context-sensitive approach

**Fig. 5:**
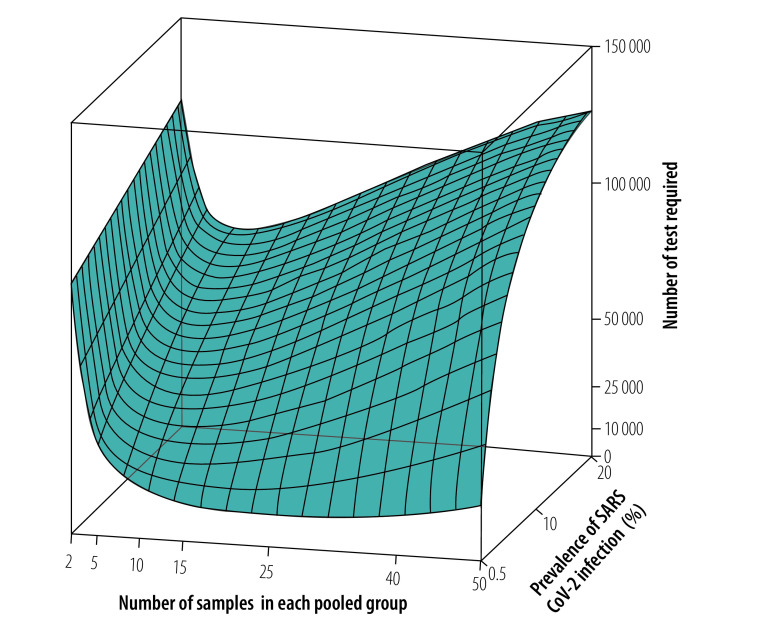
Surface plot of tests required using a context-sensitive approach to pooled-sample analysis, by pooled group size and SARS-CoV-2 infection prevalence

Our analysis of the context-sensitive approach showed that the number of tests required increased as the prevalence increased, as it did with the routine high-throughput approach. For an overall infection prevalence of 1% or 5%, the number of tests required was 58–89% less than with individual sample analysis for group sizes of 3 to 25 in a population of 150 000 (Fig. 2 and data repository).^32^ The corresponding reduction factors are shown in Fig. 3. With this low prevalence, a substantial reduction in tests required was achievable with a wide range of group sizes. For example, with a prevalence of 1%, selecting a group size between 5 and 50 implies at least 76% fewer tests. With a high prevalence of 10%, a reduction of around 65% is still achievable, though the selected group size must be close to 10 (data repository).^32^ Fig. 5 shows the number of tests required with the context-sensitive approach for a wide range of prevalences and groups sizes in a population of 150 000. The minimum number of tests required in this population was 10 740, which was achieved when the prevalence was 0.5% and the group size was 27. This result corresponded to 93% (139 260/150 000) fewer tests and a reduction factor of 14.0 compared with individual testing.

SARS-CoV-2: severe acute respiratory syndrome coronavirus 2.

Notes: The context-sensitive approach involved analysing pooled samples from groups of similar people of a defined size for real-time polymerase chain reaction testing for SARS-CoV-2. Our simulation considered a population of 150 000. Although the figure shows a continuous variation in tests required, in the simulation both prevalence and group size were varied in discrete steps.

Our sensitivity analyses confirmed that the context-sensitive approach was superior to the routine high-throughput approach for other forms of functional relationship between the within-group prevalence and the size of the pooled group (details available in the data repository).^32^ Our investigation of the scenario with a predefined mix of group sizes and a SARS-CoV-2 infection prevalence of 1% in a population of 150 000 found that 67% fewer tests would be required than with individual testing. This reduction fell between the reduction of 48% for a group size of 2 and 81% for a group size of 6.

## Discussion

We compared the effects of two pooled-sample analysis strategies on the overall number of tests required for population screening during a COVID-19 outbreak. Using Monte Carlo simulations, we found that both the routine high-throughput approach and the novel context-sensitive approach could save substantial resources during surge testing and enhanced epidemic surveillance. The routine high-throughput approach has already been proven to be cost–effective.^26^ However, the context-sensitive approach, which involves pooling samples from homogeneous groups, has a greater potential for reducing the number of tests needed for population screening.

Our simulation reflects the conditions both at the start of a general outbreak and during a second-wave outbreak in a local area where the overall prevalence of infection is low. When the prevalence in a population of 150 000 is 0.5%, the number of tests required using the context-sensitive approach varies only slightly for a wide range of group sizes. With a group size ranging from 8 to 50, between seven and 14 times fewer tests would be required compared to individual testing. Even with a group size of 5, five times fewer tests would be required. This wide range of acceptable group sizes makes this approach well suited for outbreak investigation in real-world settings. In practice, field teams could form homogenous groups of different sizes based on local conditions. 

Further, we found that even in areas with a high prevalence of around 10%, the reduction in the number of tests required would be substantial for a group size of around 10. The reduction in the numbers of tests required would also be large with the routine high-throughput approach across a wide range of group sizes in scenarios with a low infection prevalence but the number would be higher than with the context-sensitive approach. For example, if a pool size of 10 had been used in Vo' in Italy,^15^ the estimated number of tests required with the routine high-throughput approach would have been almost twice that needed with the context-sensitive approach (i.e. 1040 versus 560), assuming the within-group prevalence declined exponentially with increasing pool size.

Effectively curbing a COVID-19 outbreak involves the prompt identification and isolation of infected individuals in a short period of time.^9^^,^^15^ Curbing the outbreak is particularly important for low- and middle-income countries, where major outbreaks could exert extreme pressures on resource-poor health systems. Although widespread RT–PCR analysis provides the best method for detecting cases, individual testing will most likely not be affordable in these countries. Consequently, pooled-sample analysis could provide a better option, especially during surge testing and enhanced epidemic surveillance. Our analysis demonstrates that pooled testing could also save resources when used instead of individual testing during second-wave outbreaks, such as in Vo', where the entire population was tested and only those who tested positive were isolated.^15^

The next step in reaping the benefits of the context-sensitive pooled-sample approach is to develop implementation strategies for real-life epidemic and health systems contexts. For example, during ongoing active surveillance, several households in a specific region could be randomly selected to monitor infection prevalence and detect flare-ups early, with the specific households selected changing over time. Although some asymptomatic infected individuals could be missed, this approach would help keep the prevalence low until herd immunity is achieved or a vaccine becomes available. The timing of testing after infection is not critical because testing is ongoing and clusters can be detected on a rolling basis.

The context-sensitive approach could be implemented easily in any surveillance strategy, especially in high-income countries with civil registration systems. Individuals could be allocated to homogenous groups for pooling before field work. It may be possible to identify all symptomatic and asymptomatic individuals if a time-limited, local lockdown is in place at the time of testing. Then, only those who test positive would have to be isolated, whereas others should continue to adhere to preventive measures, such as physical distancing and wearing facemasks in enclosed public places. This approach may enable sustainable COVID-19 control without drastic population-wide measures. Although the ability of PCR-related approaches to identify all infected individuals is limited by the technique’s sensitivity and specificity, the accuracy of SARS-CoV-2 RT–PCR assays does not appear to be reduced by the use of small- or medium-sized sample pools. Moreover, recent studies on SARS-CoV-2 and other infectious agents indicate that the sensitivity and specificity of PCR assays remain high for medium-sized sample pools.^16^^,^^22^^–^^25^^,^^33^ However, additional PCR amplification cycles may be required to retain accuracy with larger sample pools.^25^

In technically well-equipped countries where high-throughput pooled PCR analysis can be performed, a multistep approach could be a good option for larger communities and cities. The testing algorithm could follow a tree structure, starting with a few large groups, such as blocks of houses, and then testing sequentially smaller groups. This approach could further reduce the number of tests required.

One strength of our simulation model is that it can be easily adjusted once more accurate estimates of disease prevalence in communities are available. We assumed that the overall prevalence is low at the beginning of an outbreak and that infections occur mainly in clusters. Later in an outbreak, infections will be spread more broadly throughout the entire population. Hence, at an early stage, a low overall prevalence is likely to be accompanied by a high within-group prevalence in a few infected groups. Our simulations captured this situation. In reality, within-group prevalence will most probably depend on the context, our model can be adjusted accordingly. For instance, for homogeneous groups formed among households in high-income countries, the within-group prevalence might decrease rapidly with group size because typical households are small and the space available per person is large. In contrast, for groups formed among larger households in densely populated areas, the slope might be flatter. The functional form of the relationship between within-group prevalence and pooled group size may be different for homogeneous groups formed from people travelling on an aircraft or working together. Our sensitivity analyses showed that our assumption of a negative exponential relationship gave a conservative estimate of the benefits of the context-specific approach; alternative relationships yielded even more favourable results (data repository).^32^ Early findings suggest that the within-group prevalence falls sharply as group size increases, though the maximum group size was limited to five in a very specific and localized high-income setting.^34^ When we assumed a steeper exponential curve, we found that the context-sensitive approach was still better at preserving resources than the routine high-throughput approach.

In conclusion, we found that a novel context-sensitive approach to pooled-sample RT–PCR screening for SARS-CoV-2 infection offered considerable potential for conserving resources during mass testing in the COVID-19 pandemic. This approach could be particularly useful for controlling outbreaks in early stages of the epidemic and during second-wave outbreaks. Countries around the world should consider adopting a context-sensitive approach as part of a sustainable, containment strategy for COVID-19.

## References

[1] Guan W-J, Ni Z-Y, Hu Y, Liang W-H, Ou C-Q, He J-X, et al. China Medical Treatment Expert Group for Covid-19. Clinical characteristics of coronavirus disease 2019 in China. N Engl J Med. 2020 4 30;382(18):1708–20. 10.1056/NEJMoa2002032 32109013 PMC7092819

[2] Lauer SA, Grantz KH, Bi Q, Jones FK, Zheng Q, Meredith HR, et al. The incubation period of coronavirus disease 2019 (COVID-19) from publicly reported confirmed cases: estimation and application. Ann Intern Med. 2020 5 5;172(9):577–82. 10.7326/M20-0504 32150748 PMC7081172

[3] Li Q, Guan X, Wu P, Wang X, Zhou L, Tong Y, et al. Early transmission dynamics in Wuhan, China, of novel coronavirus-infected pneumonia. N Engl J Med. 2020 3 26;382(13):1199–207. 10.1056/NEJMoa2001316 31995857 PMC7121484

[4] Bai Y, Yao L, Wei T, Tian F, Jin DY, Chen L, et al. Presumed asymptomatic carrier transmission of COVID-19. JAMA. 2020 2 21;323(14):1406–7. 10.1001/jama.2020.2565 32083643 PMC7042844

[5] Chan JFW, Yuan S, Kok KH, To KKW, Chu H, Yang J, et al. A familial cluster of pneumonia associated with the 2019 novel coronavirus indicating person-to-person transmission: a study of a family cluster. Lancet. 2020 2 15;395(10223):514–23. 10.1016/S0140-6736(20)30154-9 31986261 PMC7159286

[6] Lu S, Lin J, Zhang Z, Xiao L, Jiang Z, Chen J, et al. Alert for non-respiratory symptoms of coronavirus disease 2019 (COVID-19) patients in epidemic period: a case report of familial cluster with three asymptomatic COVID-19 patients. J Med Virol. 2020 3 19; 10.1002/jmv.25776 32190904

[7] Report of the WHO–China joint mission on coronavirus disease 2019 (COVID-19). Geneva: World Health Organization; 2020. Available from: https://www.who.int/publications-detail/report-of-the-who-china-joint-mission-on-coronavirus-disease-2019-(covid-19) [cited 2020 Mar 22].

[8] Cowling BJ, Aiello AE. Public health measures to slow community spread of coronavirus disease 2019. J Infect Dis. 2020 5 11;221(11):1749–51. 10.1093/infdis/jiaa123 32193550 PMC7184488

[9] Bedford J, Enria D, Giesecke J, Heymann DL, Ihekweazu C, Kobinger G, et al. WHO Strategic and Technical Advisory Group for Infectious Hazards. COVID-19: towards controlling of a pandemic. Lancet. 2020 3 28;395(10229):1015–8. 10.1016/S0140-6736(20)30673-5 32197103 PMC7270596

[10] Gilbert M, Pullano G, Pinotti F, Valdano E, Poletto C, Boëlle PY, et al. Preparedness and vulnerability of African countries against importations of COVID-19: a modelling study. Lancet. 2020 3 14;395(10227):871–7. 10.1016/S0140-6736(20)30411-6 32087820 PMC7159277

[11] Nkengasong JN, Mankoula W. Looming threat of COVID-19 infection in Africa: act collectively, and fast. Lancet. 2020 3 14;395(10227):841–2. 10.1016/S0140-6736(20)30464-5 32113508 PMC7124371

[12] Maurin M. Real-time PCR as a diagnostic tool for bacterial diseases. Expert Rev Mol Diagn. 2012 9;12(7):731–54. 10.1586/erm.12.53 23153240

[13] Fournier PE, Drancourt M, Colson P, Rolain JM, La Scola B, Raoult D. Modern clinical microbiology: new challenges and solutions. Nat Rev Microbiol. 2013 8;11(8):574–85. 10.1038/nrmicro3068 24020074 PMC7097238

[14] Chen S, Yang J, Yang W, Wang C, Bärnighausen T. COVID-19 control in China during mass population movements at New Year. Lancet. 2020 3 7;395(10226):764–6. 10.1016/S0140-6736(20)30421-9 32105609 PMC7159085

[15] Crisanti A, Cassone A. In one Italian town, we showed mass testing could eradicate the coronavirus. London: The Guardian; 2020. Available from: https://www.theguardian.com/commentisfree/2020/mar/20/eradicated-coronavirus-mass-testing-covid-19-italy-vo [cited 2020 Mar 21].

[16] Van TT, Miller J, Warshauer DM, Reisdorf E, Jernigan D, Humes R, et al. Pooling nasopharyngeal/throat swab specimens to increase testing capacity for influenza viruses by PCR. J Clin Microbiol. 2012 3;50(3):891–6. 10.1128/JCM.05631-11 22205820 PMC3295167

[17] Zhang Q, Yin Z, Li Y, Luo H, Shao Z, Gao Y, et al. Prevalence of asymptomatic *Bordetella pertussis* and *Bordetella parapertussis* infections among school children in China as determined by pooled real-time PCR: a cross-sectional study. Scand J Infect Dis. 2014 4;46(4):280–7. 10.3109/00365548.2013.878034 24520981

[18] Emmanuel JC, Bassett MT, Smith HJ, Jacobs JA. Pooling of sera for human immunodeficiency virus (HIV) testing: an economical method for use in developing countries. J Clin Pathol. 1988 5;41(5):582–5. 10.1136/jcp.41.5.582 3164325 PMC1141517

[19] Currie MJ, McNiven M, Yee T, Schiemer U, Bowden FJ. Pooling of clinical specimens prior to testing for *Chlamydia trachomatis* by PCR is accurate and cost saving. J Clin Microbiol. 2004 10;42(10):4866–7. 10.1128/JCM.42.10.4866-4867.2004 15472365 PMC522303

[20] Dorfman R. The detection of defective members of large populations. Ann Math Statist. 1943;14(4):436–40. 10.1214/aoms/1177731363

[21] Singer RS, Cooke CL, Maddox CW, Isaacson RE, Wallace RL. Use of pooled samples for the detection of Salmonella in feces by polymerase chain reaction. J Veterinary Diagn Invest. 2006 7;18(4):319–25. 10.1177/104063870601800401 16921869

[22] Ben-Ami R, Klochendler A, Seidel M, Sido T, Gurel-Gurevich O, Yassour M, et al. Large-scale implementation of pooled RNA-extraction and RT-PCR for SARS-CoV-2 detection [preprint]. Cold Spring Habor: medRxiv; 2020. Available from: https://www.medrxiv.org/content/10.1101/2020.04.17.20069062v2 [cited 2020 Mar 22]. 10.1016/j.cmi.2020.06.009 PMC7308776 32585353

[23] Edouard S, Prudent E, Gautret P, Memish ZA, Raoult D. Cost–effective pooling of DNA from nasopharyngeal swab samples for large-scale detection of bacteria by real-time PCR. J Clin Microbiol. 2015 3;53(3):1002–4. 10.1128/JCM.03609-14 25552360 PMC4390636

[24] Schmidt M, Hoehl S, Berger A, Zeichhardt H, Hourfar K, Ciesek S, et al. FACT – Frankfurt adjusted COVID-19 testing – a novel method enables high-throughput SARS-CoV-2 screening without loss of sensitivity [preprint]. Cold Spring Habor: medRxiv; 2020. Available from: https://www.medrxiv.org/content/10.1101/2020.04.28.20074187v1 [cited 2020 Mar 22].

[25] Yelin I, Aharony N, Shaer Tamar E, Argoetti A, Messer E, Berenbaum D, et al. Evaluation of COVID-19 RT-qPCR test in multi-sample pools. Clin Infect Dis. 2020 5 2;ciaa531. 10.1093/cid/ciaa531 32358960 PMC7197588

[26] Taylor SM, Juliano JJ, Trottman PA, Griffin JB, Landis SH, Kitsa P, et al. High-throughput pooling and real-time PCR-based strategy for malaria detection. J Clin Microbiol. 2010 2;48(2):512–9. 10.1128/JCM.01800-09 19940051 PMC2815636

[27] Knight GM, Dyakova E, Mookerjee S, Davies F, Brannigan ET, Otter JA, et al. Fast and expensive (PCR) or cheap and slow (culture)? A mathematical modelling study to explore screening for carbapenem resistance in UK hospitals. BMC Med. 2018 8 16;16(1):141. 10.1186/s12916-018-1117-4 30111322 PMC6094916

[28] van Hal SJ, Foo H, Blyth CC, McPhie K, Armstrong P, Sintchenko V, et al. Influenza outbreak during Sydney World Youth Day 2008: the utility of laboratory testing and case definitions on mass gathering outbreak containment. PLoS One. 2009 9 3;4(9):e6620. 10.1371/journal.pone.0006620 19727401 PMC2731881

[29] Muniesa A, Ferreira C, Fuertes H, Halaihel N, de Blas I. Estimation of the relative sensitivity of qPCR analysis using pooled samples. PLoS One. 2014 4 10;9(4):e93491. 10.1371/journal.pone.0093491 24722485 PMC3983103

[30] Thompson KM, Burmaster DE, Crouch EA. Monte Carlo techniques for quantitative uncertainty analysis in public health risk assessments. Risk Anal. 1992 3;12(1):53–63. 10.1111/j.1539-6924.1992.tb01307.x 1574617

[31] Paxton P, Curran PJ, Bollen KA, Kirby J, Chen F. Monte Carlo experiments: design and implementation. Struct Equ Modeling. 2001;8(2):287–312. 10.1207/S15328007SEM0802_7

[32] Deckert A, Bärnighausen T, Kyei NNA. Supplementary material to the publication "Pooled-sample analysis strategies for COVID-19 mass testing: a simulation study" in the WHO Bulletin. Meyrin: Zenodo; 2020. 10.5281/zenodo.3907277 10.5281/zenodo.3907277 PMC7463190 33012859

[33] Shipitsyna E, Shalepo K, Savicheva A, Unemo M, Domeika M. Pooling samples: the key to sensitive, specific and cost–effective genetic diagnosis of *Chlamydia trachomatis* in low-resource countries. Acta Derm Venereol. 2007;87(2):140–3. 10.2340/00015555-0196 17340020

[34] Streeck H, Schulte B, Kuemmerer B, Richter E, Höller T, Fuhrmann C, et al. Infection fatality rate of SARS-CoV-2 infection in a German community with a super-spreading event [preprint]. Cold Spring Habor: medRxiv; 2020. Available from: https://www.medrxiv.org/content/10.1101/2020.05.04.20090076v2 [cited 2020 Mar 22].

